# On-Call SpR Simulation Workshop for Core Trainees in Psychiatry

**DOI:** 10.1192/bjo.2026.11281

**Published:** 2026-06-30

**Authors:** Leopold Rudolph

**Affiliations:** Oxleas NHS Foundation Trust, London, United Kingdom

## Abstract

**Aims:**

Simulation training is a type of experiential learning where individuals practice skills and decision-making in a safe, artificial environment that mirrors real-world scenarios.Transition points in medical careers, such as the step up from Core to Specialty Training in Psychiatry, are often viewed in a negative anticipatory fashion by doctors about to undertake a role change. A simulated workshop was created, with the aim to support Core Psychiatry Trainees (CTs) to develop their confidence in acting as the Specialty Registrar (SpR).

**Methods:**

A pilot session was delivered at an Oxleas local Core Trainee teaching day. CTs were requested to bring cases that they had previously discussed with a SpR when on-call. 20 trainees in attendance were split into four groups, each with a Registrar/Consultant facilitator. In each group, the more senior CTs were asked to role play as the SpR, with the junior CTs presenting their cases to them. Feedback was provided on the content and communication skills of the CTs discussing their cases, and on the management plans of the CTs acting as the SpR. Additionally, to add to the fidelity of the simulation, vignettes of common scenarios requiring SpR input out-of-hours had been pre-prepared and the CTs taking on the SpR role were also asked to provide their advice on these situations.

**Results:**

A QR code for a survey was displayed immediately after the session finished. 15 Core Trainees responded (~75% completion rate), with all CTs reporting that the workshop was useful and engaging. Moreover, there was a considerable increase in trainee confidence of taking on the role of a SpR following the simulation, although final confidence levels remained mixed. Participants fed-back that they liked the concept, the structure and interactiveness of the session, as well as the realism of the scenarios. Feedback from the facilitators also echoed that of the CTs.

**Conclusion:**

This was a successful pilot workshop utilising simulation to increase the confidence of Core Psychiatry Trainees in stepping up to the role of a SpR. Ideas forimprovement include the addition of scenarios illustrating dilemmas regarding the use of the Mental Health Act, as well as the option of a debrief between CTs and SpRs following the class where any on-call concerns can be discussed. Given the strong positive response, further work will be undertaken to refine the simulation session, with the ambition to continue its delivery locally, alongside rolling it out regionally.

